# Supplementary material for: Immune Response Against Influenza in a Cohort of Repeatedly Vaccinated Adults During the 2017/2018 and 2018/2019 Seasons
Source: Vaccines (Basel). 2024 Oct 26;12(11):1218. doi: 10.3390/vaccines12111218 (PMC11598577; doi:10.3390/vaccines12111218)
Supplement: Supplementary file 1 [file vaccines-12-01218-s001.zip › vaccines-3214600-supplementary.pdf]

Supplementary material

Table S1. Influenza vaccination profile of the HCW classified as frequently (≥ 3 vaccines) and occasionally (≤ 2 vaccines) vaccinated, recruited in 2017/2018 and 2018/2019 seasons.

Categories	Vac_2015/2016	Vac_2016/2017	Vac_2017/2018	Vac_2018/2019
2017_Occasional		V	V	
2017_Frequent	V	V	V	
2017_Occasional			V	
2017_Occasional	V		V	
2018_Frequent		V	V	V
2018_Frequent	V	V	V	V
2018_Occasional			V	V
2018_Frequent	V		V	V
2018_Occasional				V
2018_Occasional		V		V

Table S2. Demographic characteristics and vaccination history of the participants included in the follow-up in both seasons, 2017/2018 and 2018/2019 (N=49). Participants frequently and occasionally vaccinated were categorized at recruitment.

			**Vaccination n (%)**		
		**Total n (%)**	Frequent (≥ 3 vaccines)	Occasional (≤ 2 vaccines)	2009 pandemic vaccine
**2017/2018 and 2018/2019**	N	49	20	29	15
	Age group (yr) n (%)				
	20-49	23 (46.9%)	9 (45.0%)	14 (48.3%)	8 (53.3%)
	50-69	26 (53.1%)	11 (55.0%)	15 (51.7%)	7 (46.7%)
	Mean (SD)	50.4 (8.3)	50.1 (9.0)	50.6 (7.9)	50.0 (6.9)
	Range	34 - 69	34 - 68	35 - 69	34 - 60
	Sex n (%)				
	Female	36 (73.5%)	13 (65.0%)	23 (79.3%)	10 (66.7%)
	Male	13 (26.5%)	7 (35.0%)	6 (20.7%)	5 (33.3%)
	Chronic disease n (%)				
	No	38 (77.6%)	16 (80.0%)	22 (75.9%)	12 (80.0%)
	Yes	11 (22.4%)	4 (20.0%)	7 (24.1%)	3 (20.0%)
	ILI n (%)				
	Influenza positive	1 (12.5%)	0 (0.0%)	1 (12.5%)	1 (50.0%)
	Negative	7 (87.5%)	0 (0.0%)	7 (87.5%)	1 (50.0%)

Table S3. Immunological response to seasonal trivalent influenza vaccine during 2017/2018 and 2018/2019 for frequently and occasionally vaccinated individuals. Antibodies against influenza B Yamagata.

	**Influenza B Yamagata***			
	Total	Frequent vacc	Ocasional vacc	Ratio
**2017/2018 (N)**	74	32	42	
**Seroprotection rate % (95% CI)**				
**Prevaccination**	4.1 (0.8;11.4)	6.3 (0.8;20.8)	2.4 (0.1;12.6)	0.4 (0;4.0)
**1 month postvaccination**	24.3 (15.1;35.7)	28.1 (13.7;46.7)	21.4 (10.3;36.8)	0.8 (0.3;1.7)
**6 months postvaccination**	37 (26;49.1)	50.0 (31.9;68.1)	26.8 (14.2;42.9)	0.5 (0.3;1)

**Seroconversion rate % (95% CI)**				
**1 month postvaccination**	18.9(10.7;29.7)	18.8(7.2;36.4)	19.0(8.6;34.1)	1.0 (0.4;2.6)
**6 months postvaccination**	23 (14;34.2)	28.1 (13.7;46.7)	19.0(8.6;34.1)	0.7 (0.3;1.6)
**GMT (95%CI)**				
**Prevaccination**	8.4 (7.3;9.8)	11.6 (9.3;14.6)	6.6 (5.6;7.8)	0.6 (0.2;1.5)
**1 month post-accination**	12.5 (10.1;15.5)	16.5 (12;22.5)	10.2 (7.7;13.5)	0.6 (0.3;1.4)
**6 months postvaccination**	25.4 (21.3;30.2)	32.2 (25.1;41.3)	21 (16.6;26.7)	0.7 (0.4;1.1)
**GMT ratio (95%CI)**				
**1 month postvaccination**	1.5 (1.3;1.8)	1.4 (1.1; 1.8)	1.5 (1.2; 1.9)	
**6 months postvaccination**	3.0 (2.6; 3.6)	2.8 (2.1; 3.6)	3.2 (2.6; 4.0)	
**2018/2019 (N)**	70	41	29	
**Seroprotection rate % (95% CI)**				
**Prevaccination**	28.2 (18.1;40.1)	37.2 (23;53.3)	14.3 (4;32.7)	0.4 (0.1;1)
**1 month postvaccination**	35.2 (24.2;47.5)	46.5 (31.2;62.3)	17.9 (6.1;36.9)	0.4 (0.2;0.9)
**6 months postvaccination**	31.9 (21.2;44.2)	42.9 (27.7;59)	14.8 (4.2;33.7)	0.3 (0.1;0.9)
**Seroconversion rate % (95% CI)**				
**1 month postvaccination**	4.2 (0.9;11.9)	7.0 (1.5;19.1)	0.0 (0;11.9)	0.0
**6 months postvaccination**	1.4 (0;7.5)	2.3 (0.1;12.3)	0.0 (0;11.9)	0.0
**GMT (95%CI)**				
**Pre-vaccination**	17.1 (14;20.9)	21.3 (16.5;27.6)	12.2 (9;16.5)	0.6 (0.3;1.2)
**1 month postvaccination**	20.4 (16.5;25.3)	26.3 (19.9;34.8)	13.8 (10.3;18.6)	0.5 (0.3;1.0)
**6 months postvaccination**	18.3 (14.9;22.5)	22.4 (17.3;29.1)	13.3 (9.9;17.8)	0.6 (0.3;1.2)
**GMT ratio (95%CI)**				
**1 month postvaccination**	1.2 (1.1; 1.3)	1.2 (1.1; 1.4)	1.1 (1.0; 1.2)	
**6 months postvaccination**	1.1 (1.0;1.1)	1.1 (1.0; 1.2)	1.1 (1.0; 1.1)	

* B Yamagata was not included in influenza vaccine composition for 2017/2018 and 2018/2019 seasons.

Table S4. GMT and GMT ratio (GMT R) for occasionally vs. frequently vaccinated HCW, adjusted for age and gender, for influenza A(H1N1), A(H3N2), B Victoria, B Yamagata, during the follow-up of 49 participants that repeatedly uptake seasonal TIV during 2017/2018 and 2018/2019 seasons.

				GMT and GMT ratio for occasionally vs. frequently vaccinated HCW							
**Season**				AH1N1		AH3N2		B Vitoria		B Yamagata	
				GMT (CI 95%)	GMT R * (CI 95%)	GMT (CI 95%)	GMT R * (CI 95%)	GMT (CI 95%)	GMT R * (CI 95%)	GMT (CI 95%)	GMT R * (CI 95%)
**2017/18**	Prevaccination	S0	Frequent vacc	16.2 (11.3; 23.4)	-	20 (13.0; 30.8)	-	23.8 (16.3;34.7)	-	23.8 (16.3; 34.7)	Ref.
			Ocasional vacc	22 (15.7; 30.8)	1.4 (0.9; 2.2)	27.3 (19.4; 38.4)	1.3 (0.8; 2.1)	16.9 (13.3;21.5)	0.7 (0.5-1.0)	16.9 (13.3; 21.5)	0.6 (0.4-0.7)
	1 month postvaccination	S0+1m	Frequent vacc	22.2 (15.4; 32.0)	-	33.6 (22.1; 51.1)	-	24.6 (16.8;36)	Ref.	24.6 (16.8; 36.0)	Ref.
			Ocasional vacc	47.3 (31.1; 72.0)	2.2 (1.3; 3.7)	61.5 (44.4; 85.2)	1.7 (1.1; 2.6)	33 (25.2;43.3)	1.4 (0.9-2.1)	33 (25.2; 43.3)	0.7 (0.4-1.2)
	6 months postvaccination	S0+6m	Frequent vacc	12.7 (9.4; 17.2)	-	24.6 (17.4; 34.9)	-	18.7 (13.6;25.5)	Ref.	18.7 (13.6; 25.5)	Ref.
			Ocasional vacc	16 (12.4; 20.6)	1.3 (0.8; 1.9)	31.2 (23.5; 41.5)	1.1 (0.7; 1.9)	20.5 (15.7;26.8)	1.2 (0.8-1.8)	20.5 (15.7; 26.8)	0.9 (0.6-1.2)
**2018/19**	Prevaccination	S0+12m	Frequent vacc	18.2 (14.9; 22.2)	-	21.6 (15.8; 29.4)	-	17.2 (13.5;22)	Ref.	17.2 (13.5; 22.0)	Ref.
			Ocasional vacc	22.4 (16.4; 30.7)	1.2 (0.9; 1.7)	35.6 (20.5; 61.9)	1.3 (0.7; 2.3)	11.3 (7.2;17.8)	0.7 (0.4-1.0)	11.3 (7.2; 17.8)	0.6 (0.4-0.9)
	1 month postvaccination	S0+13m	Frequent vacc	41.5 (33.0; 52.3)	-	67.6 (47.4; 96.3)	-	37.8 (29.2;48.9)	Ref.	37.8 (29.2; 48.9)	Ref.
			Ocasional vacc	47.6 (29.0; 78.1)	1.2 (0.7; 1.9)	100.8 (60.9; 166.7)	1.3 (0.8; 2.3)	21.3 (13.2;34.4)	0. 6 (0.4-0.9)	21.3 (13.2; 34.4)	0.5 (0.3-0.8)
	6 months postvaccination	S0+18m	Frequent vacc	20.4 (16.7; 24.9)	-	36.3 (25.3; 52.3)	-	26.2 (20.1;34.1)	Ref.	26.2 (20.1; 34.1)	Ref.
			Ocasional vacc	17.6 (11.2; 27.7)	0.9 (0.6; 1.4)	70.5 (42.9; 115.8)	1.7 (1.1; 2.9)	20 (13.4;29.8)	0.7 (0.5-1.1)	20 (13.4; 29.8)	0.5 (0.3-0.9)

*Generalized estimating equations model adjusted for age, sex and presence of chronic disease

Text S1. Influenza vaccine composition for the northern hemisphere influenza seasons, 2017/2018 and 2018/2019.

The influenza 2017/2018 trivalent vaccine composition (inactivated surface antigen, 15micrograms HA): A/Michigan/45/2015 (H1N1)pdm09-like virus; an A/Hong Kong/4801/2014 (H3N2)-like virus; and B/Brisbane/60/2008-like virus.

The influenza 2018/2019 trivalent vaccine composition (inactivated surface antigen, 15micrograms HA): A/Michigan/45/2015(H1N1)pdm09-like virus; an A/Singapore/INFIMH-16-0019/2016(H3N2)-like virus; a B/Colorado/06/2017-like virus (B/Victoria/2/87 lineage);

The influenza A(H1N1)pdm09 monovalent pandemic vaccine composition: AS03-adjuvanted split virus vaccine, containing 3.75 µg hemagglutinin of A/California/7/2009(H1N1)pdm09.

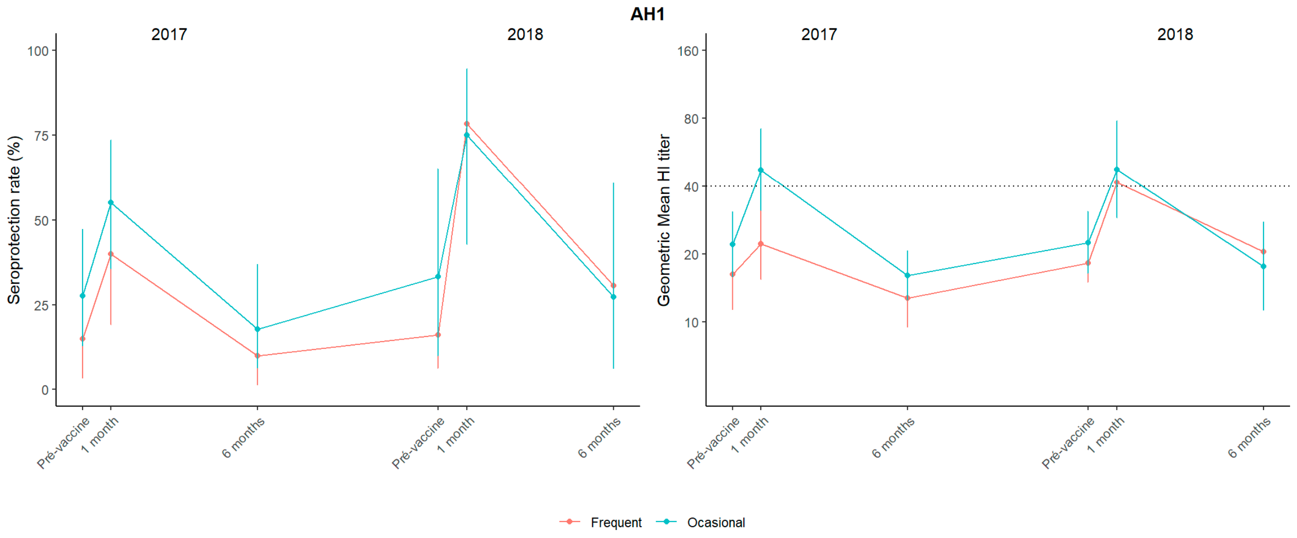

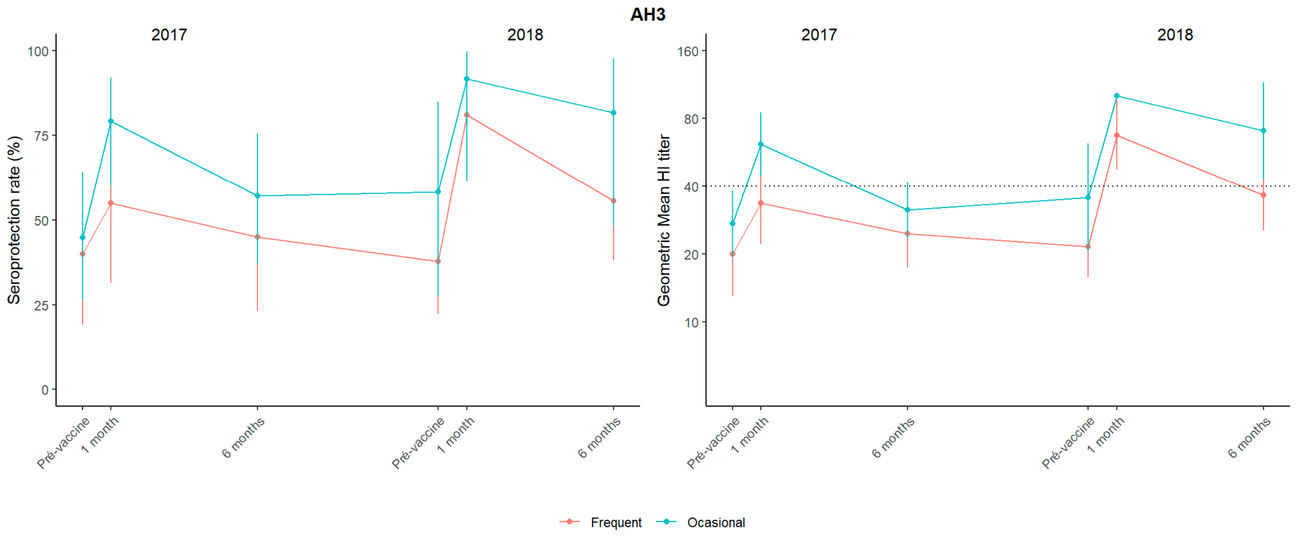

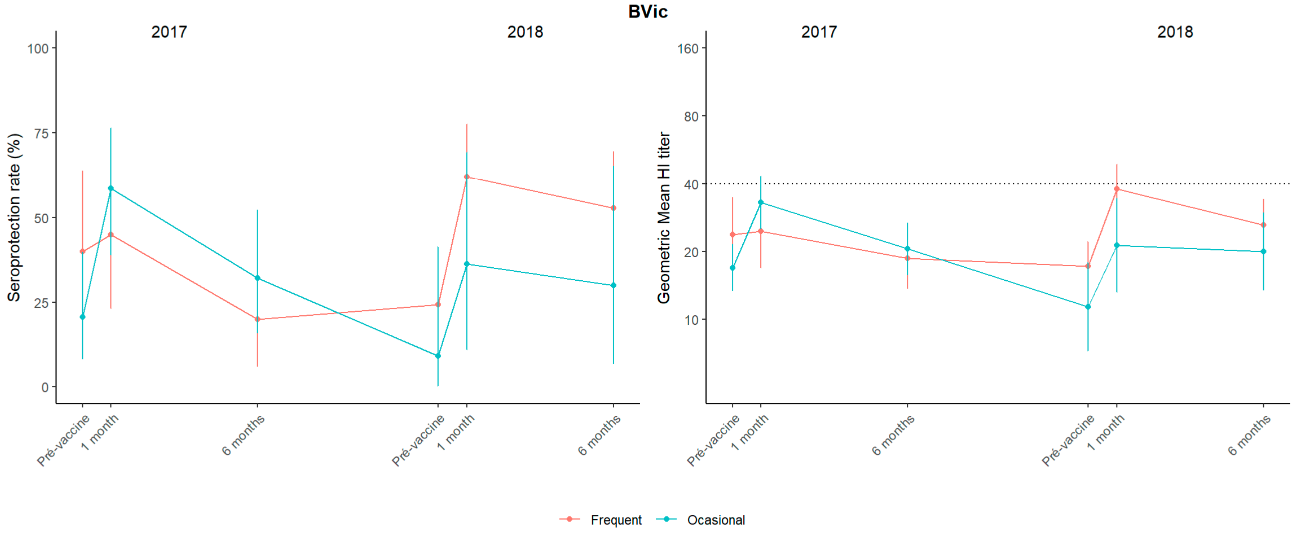

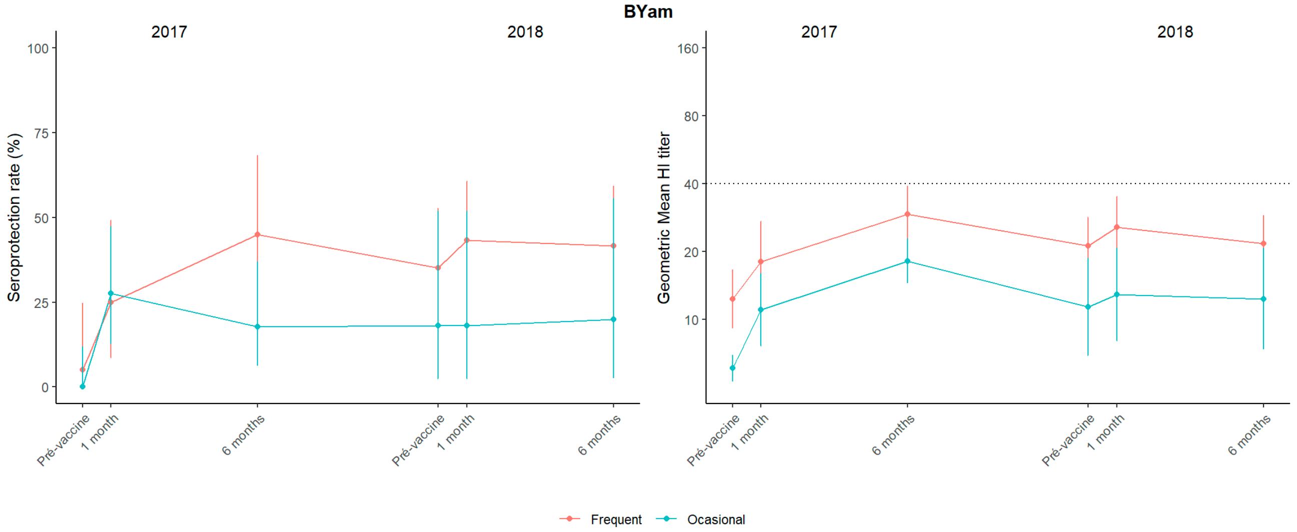

Figure S1 Seroprevalence and GMT of protective antibodies against influenza A subtypes and influenza B lineages after repeated annual TIV vaccination during 2017/2018 and 2018/2019 seasons (n=49) for frequently (≥ 3 TIV) and occasionally vaccinated (≤ 2 TIV) HCW for the 6 time points during the follow-up period (pre-vaccine 2027/2028, 1 and 6 months post vaccination, pre-vaccine 2018/2019 and 1 and 6 months post vaccination) .
